# Editorial: Collection on cochlear implantation and speech perception

**DOI:** 10.3389/fnhum.2023.1344875

**Published:** 2024-01-04

**Authors:** Celine Richard, Nancy M. Young

**Affiliations:** ^1^Department of Otolaryngology-Head and Neck Surgery, University of Tennessee Health Science Center, Memphis, TN, United States; ^2^Division of Otolaryngology-Head and Neck Surgery, Lebonheur Children's Hospital, Memphis, TN, United States; ^3^Division of Otolaryngology-Head and Neck Surgery, St. Jude Children's Research Hospital, Memphis, TN, United States; ^4^Department of Otolaryngology-Head and Neck Surgery, Feinberg School of Medicine, Northwestern University, Chicago, IL, United States; ^5^Division of Otolaryngology-Head and Neck Surgery, Ann and Robert H. Lurie Children's Hospital of Chicago, Chicago, IL, United States

**Keywords:** cochlear implant, speech perception, evoked compound action potentials (ECAP), listening efficiency, single sided deafness

The primary objective in the scientific and medical community is to achieve high-level speech perception in cochlear implant (CI) recipients. This pursuit is underpinned by a multitude of published studies and research efforts dedicated to advancing cochlear implant technology and clinical practice.

Cochlear implantation enhances hearing for those with significant sensorineural hearing loss not adequately remediated by amplification. However, there is considerable variability in speech perception and spoken language outcome (Sarant et al., 2001; Boisvert et al., 2020; Heutink et al., 2021). Variables such as the cause of hearing loss, duration of deafness, the integrity of the interface between the implanted electrode array and the auditory nerve, differences in implant devices and programming, and individual neural plasticity contribute to the diversity in outcomes.

In their article, Liebscher et al. put forth the hypothesis that preoperative residual hearing might influence the electrically evoked compound action potentials (ECAP) observed after cochlear implantation. To explore this hypothesis, they conducted a retrospective analysis on a cohort of CI recipients, measured their intraoperative ECAP responses and estimated spiral ganglion frequency by analyzing the specific positions of the electrode contacts, as determined from postoperative CT scans. They observed that individuals with better preoperative hearing exhibited lower ECAP thresholds, irrespective of the hearing preservation status of their hearing. Additionally, recipients with preserved residual hearing had lower intraoperative ECAP thresholds at apical electrodes.

Two articles centered their attention on the heightened listening effort experienced in real-world listening conditions, employing either behavioral measures (Perea Pérez et al.) or objective testing (Xiu et al.).

Perea Pérez et al. introduced an innovative method for quantifying “listening efficiency,” a comprehensive metric that accounts for both task performance and response time in auditory tasks. This method tracks the rate of evidence accumulation toward correct responses within a linear ballistic accumulator (LBA) model of decision-making. This proposed listening efficiency metric holds promise as an outcome measure to characterize the speed-accuracy trade-off in participants' performance under challenging listening conditions. It exhibited sensitivity to changes in task demands and discerned significant group differences. Specifically, within the CI group, listening efficiency showed moderate-to-strong correlations with cognitive and self-reported measures of listening effort. Further research is warranted to explore the sensitivity and practical applicability of the authors listening efficiency metric across diverse listening scenarios.

On the other hand, Xiu et al. underscored the constraints of contemporary clinical assessments for CI recipients, highlighting a tenuous association between clinical evaluations and self-reported speech perception outcomes. Real-world speech comprehension is influenced by many factors, including visual cues, ambient acoustic conditions, and the conversational context, all of which exert substantial influence on listening demand. Their investigation used continuous electroencephalography to quantify neural tracking of speech and evaluate the associated listening demand. The results reveal that heightened noise levels are linked to increased subjective mental demand and diminished word/conversation comprehension in cochlear implant users. Furthermore, increased background noise was found to increase perceived cognitive demand during noise-laden listening tasks, whereas visual cues play a more complex role for CI users in higher-noise conditions. These findings support the development of objective measures to gauge listening effort and cognitive fatigue in CI users during realistic scenarios. This approach may be advantageous for young children and others unable to be assessed by traditional task-based measures.

In their article, Warren and Atcherson examined selective electrode deactivation to improve speech perception when the electrode-neural interface is not optimal. Clinical audiologists typically deactivate electrodes in response to specific issues like abnormal telemetry data, extracochlear electrode indications, or facial stimulation. Deactivation of electrodes to improve pitch ranking is not standard practice because evidence that is approach is beneficial is limited and standardized deactivation protocols had not been available. The study provides a strategy for identifying electrodes likely to contribute to the degradation of pitch perception, enabling the deactivation of specific electrodes during pitch ranking assessment. The experimental program improved speech perception and the resulting map was preferred by most adult CI users. These findings can guide the development of future programming methods for cochlear implants by audiologists.

Van Bogaert et al. conducted a study that investigated the supportive efficacy of two separate speech rehabilitation methodologies for pediatric cochlear implant recipients: the multisensory technique, recognized as French Cued Speech, and the auditory-centric methodology of Auditory Verbal Therapy (AVT). The authors found that both Cued Speech and AVT can bolster speech perception. This study highlights the significance of implementing effective therapies to enhance speech perception in children with cochlear implants, especially during the early post-implantation years. It underscores the crucial role of parental involvement in a child's rehabilitation, as both methods heavily depend on active parental engagement.

In their article focused on pediatric CI recipients with single-sided deafness (SSD), Park et al. examined time-related factors that may affect word recognition in the CI ear alone and spatial release from masking (SRM). They found better word recognition in pediatric SSD CI users with longer duration of device use since activation, higher daily CI use, and shorter duration of deafness. The age at implantation did not significantly predict Consonant-Nucleus-Consonant word recognition. Interestingly, the Hearing Hours Percentage metric (HHP) defined as the amount of time CI users have access to sound as compared to their typically hearing peers, and age at activation were the only time factors associated with the amount of SRM experienced by these children. While their research underscores the importance of duration of deafness on speech perception outcomes, the authors support implantation of children with longer duration of deafness in light of the benefits that still may be achieved, especially if consistent device use over a longer time frame. They highlight the pivotal role of effective preoperative counseling to convey the range of benefits and significance of device use to patients and their families before surgery.

This editorial emphasizes the complex relationship between various factors, such as speech rehabilitation, cochlear implant mapping, electrode optimization, preoperative counseling, and family involvement, in influencing speech perception outcomes in CI recipients.

## Author contributions

CR: Writing—original draft, Writing—review & editing. NY: Writing—original draft, Writing—review & editing.
